# Ventricular Repolarization Abnormalities in Pediatric Athletes: A Practical Approach to Clinical Evaluation

**DOI:** 10.3390/jcdd13050185

**Published:** 2026-04-28

**Authors:** Lorenzo Morra, Riccardo Borzuola, Antonio Gianfelici, Francesco Nifosì, Federico Quaranta, Leonardo Calò, Fabio Pigozzi, Chiara Fossati

**Affiliations:** 1 Department of Movement, Human and Health Sciences, University of Rome Foro Italico, 00135 Rome, Italy; l.morra@studenti.uniroma4.it (L.M.); a.gianfelici@studenti.uniroma4.it (A.G.); federico.quaranta@uniroma4.it (F.Q.); leonardo.calo@uniroma4.it (L.C.); fabio.pigozzi@uniroma4.it (F.P.); chiara.fossati@uniroma4.it (C.F.); 2 Centre for Exercise Science and Sports Medicine, University Foundation Foro Italico, 00135 Rome, Italy; francesconifosi92@gmail.com; 3 Italian Federation of Sports Medicine-AMS Roma, 00196 Rome, Italy; 4 Department of Cardiology, Policlinico Casilino, 00169 Rome, Italy

**Keywords:** pediatric athletes, adolescent athletes, ventricular repolarization, early repolarization pattern, T-wave inversion, electrocardiography, sports cardiology, sudden cardiac death, risk stratification, cardiac magnetic resonance imaging

## Abstract

Ventricular repolarization abnormalities are among the most frequent electrocardiographic findings in pediatric athletes undergoing cardiovascular screening, yet their clinical significance remains a major source of diagnostic uncertainty. While most of them represent benign expressions of training-induced cardiac remodeling and developmental maturation, selected patterns may constitute the earliest phenotypic manifestation of cardiomyopathies or primary electrical disease. Distinguishing physiological adaptation from early pathology is therefore essential to prevent both sudden cardiac events and unnecessary restrictions on sports participation. This review integrates contemporary international electrocardiographic interpretation criteria with emerging pediatric evidence to provide a clinically oriented framework for evaluation and risk stratification of ventricular repolarization abnormalities in pediatric athletes. Early repolarization and anterior T-wave inversion are commonly benign when occurring within recognized age- and ethnicity-specific patterns and in the absence of symptoms, concerning family history, or structural abnormalities. Conversely, lateral or inferolateral T-wave inversion, atypical ST-segment morphology, complex ventricular arrhythmias, and abnormal imaging findings represent red flags requiring comprehensive investigation, including multimodality imaging when indicated. Due to the dynamic electrophysiological evolution during adolescence, longitudinal reassessment is crucial. A structured, risk-based approach integrating electrocardiographic features, demographic/familial context, clinical evaluation, imaging findings, and follow-up provides a pragmatic strategy to optimize risk detection while safeguarding appropriate athletic participation in young athletes.

## 1. Introduction

Sudden cardiac death (SCD) in young athletes, although uncommon, remains a dramatic event with profound clinical and social implications. Epidemiological data estimate the incidence of SCD in competitive athletes to range between one and three cases per 100,000 athletes per year, with variability according to age, sex, and geographic region [1,2]. Structural cardiomyopathies and channelopathies/electrical diseases represent the leading causes in this population, with hypertrophic cardiomyopathy and arrhythmogenic cardiomyopathy being particularly prominent substrates [2,3].

Pre-participation cardiovascular screening aims to identify individuals at risk before catastrophic events occur. Corrado et al. demonstrated that inclusion of a resting 12-lead electrocardiogram (ECG) significantly reduced the incidence of SCD in athletes [4]. Later investigations confirmed that ECG increases sensitivity in detecting cardiomyopathies and channelopathies compared to the collection of family history and physical examination alone [5].

However, ECG interpretation in athletes presents substantial challenges. Chronic exposure to intensive training induces structural and electrical remodeling—commonly referred to as “athlete’s heart”—which may include sinus bradycardia, increased QRS voltages, and repolarization changes that can overlap with pathological findings [6]. Ventricular repolarization abnormalities (VRAs), including early repolarization pattern (ERP), T-wave inversion (TWI), ST-segment deviations, and QT interval alterations, are particularly frequent and complex to diagnose in athlete populations [7].

The development of standardized ECG interpretation criteria, culminating in current international recommendations, has substantially improved diagnostic specificity while maintaining high sensitivity for clinically relevant disease [7,8]. Nevertheless, interpretation becomes more complex in pediatric athletes. As a matter of fact, age-dependent electrophysiological maturation influences ECG morphology, and anterior T-wave inversion may represent a physiological juvenile pattern in younger individuals, often regressing with puberty [9].

Despite growing consensus on ECG interpretation, uncertainties remain regarding the long-term prognostic significance of isolated ventricular repolarization abnormalities in asymptomatic pediatric athletes. Emerging longitudinal data suggest that, while most repolarization patterns are benign expressions of training adaptation, a minority may represent early phenotypic expression of cardiomyopathy, warranting structured evaluation and follow-up [10].

This review focuses on ventricular repolarization abnormalities in pediatric athletes, integrating current evidence on epidemiology, pathophysiology, clinical interpretation, and risk stratification to provide a practical framework for clinicians involved in the cardiovascular care of young athletes.

## 2. Literature Search Strategy

This review was developed through a structured search of the PubMed/MEDLINE database, focusing on studies published in English addressing ventricular repolarization abnormalities in pediatric and adolescent athletes. Search terms included combinations of “athlete,” “pediatric,” “adolescent,” “electrocardiogram,” “ventricular repolarization,” “early repolarization,” “T-wave inversion,” and “sports cardiology”.

Priority was given to original research studies, longitudinal cohort analyses, consensus statements, and international guidelines relevant to ECG interpretation and clinical management in young athletic populations. Additional studies were identified through manual screening of bibliographies of key articles.

The literature search was limited to scientific studies in English published between January 2009 and December 2026.

## 3. International ECG Recommendations and Age-Specific Interpretation in Pediatric Athletes

The interpretation of electrocardiographic findings in athletes has evolved substantially over the past two decades. Early European recommendations represented the first structured attempt to standardize ECG interpretation and reduce false-positive findings in screening programs [11]. More recent refinements, including the Seattle Criteria, significantly improved specificity while preserving sensitivity for detection of clinically relevant cardiovascular disease [12].

The development of updated international recommendations further harmonized ECG interpretation across different cohorts of athletes [13]. The 2017 International Criteria for Electrocardiographic Interpretation in Athletes represented a major milestone, demonstrating a marked reduction in abnormal ECG rates compared with earlier standards while maintaining high sensitivity for cardiomyopathies and channelopathies associated with SCD [13]. Application of current recommendations has reduced abnormal ECG rates to below 5% in most screened cohorts [12,13].

The 2017 International Criteria classify ECG findings into normal (training-related), borderline, and abnormal categories. Borderline findings require contextual interpretation, particularly when more than one are evidenced, whereas abnormal findings mandate further evaluation with imaging and functional testing [13].

In pediatric athletes, ECG interpretation should consider age-dependent electrophysiological maturation. Anterior T-wave inversion in leads V1–V3 may represent a physiological juvenile pattern in athletes aged under 16 and frequently regresses during pubertal development [9]. Persistence beyond mid-adolescence (≥16 years of age) or extension beyond lead V2 increases suspicion for underlying structural disease [14].

The 2020 ESC Guidelines on sports cardiology emphasize a structured approach integrating ECG morphology with symptoms, family history, and targeted multimodality imaging when indicated [15]. In pediatric populations, longitudinal reassessment is particularly important due to the dynamic nature of cardiac maturation during adolescence [15].

Furthermore, ethnicity must be considered as it plays a critical role in ECG interpretation. For instance, studies in elite Black athletes demonstrated that anterior T-wave inversion preceded by J-point elevation and convex ST-segment elevation may represent a physiological variant rather than pathology [8]. Recognition of ethnicity-related patterns has substantially reduced unnecessary investigations and inappropriate restriction from competitive sport [8,16].

## 4. Physiological Mechanisms Underlying Ventricular Repolarization Abnormalities in Athletes

Regular intensive training induces structural and electrical adaptations collectively known as “athlete’s heart.” These adaptations reflect physiological remodeling driven by chronic hemodynamic loading conditions [17]. Cardiac remodeling includes increased ventricular cavity dimensions and mild wall thickening within the physiological range, potentially influencing ECG morphology [17,18].

Enhanced parasympathetic tone in trained individuals contributes to resting sinus bradycardia and modulation of ventricular repolarization dynamics [19]. Increased vagal activity may favor J-point elevation and ST-segment patterns typical of ERP [19,20].

ERP has been linked to increased epicardial action potential notch mediated by transient outward potassium current, contributing to transmural dispersion of repolarization [21]. Although similar mechanisms underlie malignant J-wave syndromes, ERP in athletes is generally associated with benign autonomic and structural adaptation rather than arrhythmogenic risk [21,22].

ST-segment morphology following J-point elevation is clinically relevant. Horizontal or descending ST segments have been associated with increased arrhythmic risk in non-athletic populations, whereas rapidly ascending ST segments—common in athletes—are considered benign [22,23].

Endurance training may induce right ventricular remodeling, potentially influencing repolarization patterns, including anterior repolarization variants such as T-wave inversion in leads V1–V3 or flattened T-waves in the right precordial leads, as well as other ECG findings suggestive of right ventricular adaptation, such as right axis deviation, incomplete or complete right bundle branch block, and increased R-wave voltage in leads V1–V2. These ECG patterns should be interpreted for a differential diagnosis between physiological remodeling and arrhythmogenic cardiomyopathy [24]. However, the absence of ventricular dysfunction or fibrosis could point toward a physiological adaptation [25].

In young athletes, exercise adaptation overlaps with pubertal maturation of the cardiovascular system. Developmental changes in ion channel expression and autonomic balance influence ECG morphology during adolescence [26]. Hormonal modulation may partially explain sex-related differences in ERP prevalence and ST-segment patterns observed in young athletes [26]. Supporting the potential role of hormonal modulation during adolescence, Pieles et al. reported a significantly higher prevalence of early repolarization in male athletes compared with females, suggesting that sex-related electrophysiological differences emerging during pubertal maturation may contribute to the expression of this ECG pattern [27]. Consistent with these findings, Sinner et al. demonstrated that both sex and age significantly influence the prevalence of early repolarization, with a higher frequency observed in younger individuals and in males, highlighting the contribution of biological and maturational factors to the development of this repolarization phenotype [28]. Similarly, Patton et al. emphasized the presence of important sex-related differences in ventricular repolarization, noting that early repolarization patterns are more frequently observed in males and may reflect underlying biological mechanisms influencing cardiac electrophysiology [26].

## 5. Early Repolarization Pattern in Pediatric Athletes

### 5.1. Definition and ECG Characteristics of ERP

ERP is defined by a J-point elevation ≥ 0.1 mV in at least two contiguous inferior and/or lateral leads, often accompanied by terminal QRS notching or slurring and variable ST-segment morphology. In athletes, ERP is typically considered a training-related finding when associated with ascending ST segments [13,29].

Interest in ERP has increased following reports linking inferolateral ERP with horizontal or descending ST segments to idiopathic ventricular fibrillation in non-athletic populations [22]. Contemporary interpretation therefore emphasizes both lead distribution and ST morphology [30]. In athletes, ERP most commonly involves the inferior and lateral leads, although variations in distribution may occur depending on age, training load, and autonomic balance. The pattern is typically characterized by a distinct J-point elevation with either notching or slurring of the terminal portion of the QRS complex, followed by an ST segment that usually demonstrates a rapidly ascending morphology. In highly trained individuals, ERP may appear more prominent during periods of increased vagal tone, particularly at rest or during sleep. The amplitude of J-point elevation can vary over time and may fluctuate with training intensity, hydration status, and heart rate. These dynamic features highlight the importance of interpreting ERP within the broader physiological context of athletic adaptation [31].

### 5.2. Prevalence and Age-Related Patterns

ERP is relatively frequent in pediatric and adolescent athletes and may evolve over time. In screening cohorts of athletes ≤ 16 years, ERP prevalence has been reported to be around 13%, predominantly involving inferolateral leads with no adverse cardiovascular outcomes during follow-up [32].

In adolescents, ERP prevalence may approach 25%, with sex-related differences in ST morphology but without increased arrhythmic burden during maximal exercise testing [29]. The prevalence of ERP may also vary according to training intensity and the type of sport, with endurance disciplines generally showing a higher frequency of this pattern compared with skill-based or power sports [32]. In addition, ERP expression in young athletes often demonstrates a dynamic behavior over time, with changes in J-point amplitude or ST-segment morphology observed during longitudinal ECG evaluations [32,33]. These findings support the concept that ERP in pediatric and adolescent athletes represents a modifiable electrophysiological phenotype influenced by both physiological maturation and ongoing training adaptation. Representative studies evaluating the prevalence and characteristics of ERP in pediatric athletes are summarized in Table 1.

### 5.3. Association with Cardiac Remodeling

ERP frequently coexists with physiological remodeling. Studies in teenage competitive athletes demonstrated association between ERP and benign left ventricular remodeling without structural pathology on imaging [33]. This association likely reflects the shared physiological mechanisms underlying electrical and structural adaptation to regular training. Increased left ventricular cavity size and enhanced diastolic function, commonly observed in trained adolescents, may contribute to the electrophysiological substrate that favors the expression of ERP on surface ECG. In this context, ERP should be interpreted as part of the broader spectrum of exercise-induced cardiac remodeling rather than as an isolated electrical phenomenon. Importantly, when structural evaluation demonstrates normal cardiac morphology and function, the coexistence of ERP and physiological remodeling is generally considered a benign finding [33].

### 5.4. Prognostic Implications

Available data suggest that isolated ERP in pediatric athletes—particularly with ascending ST morphology—most often represents a benign training-related finding. Prospective pediatric cohorts show absence of adverse events during medium-term follow-up [32]. Accordingly, ERP should prompt contextual evaluation rather than automatic escalation to advanced testing in asymptomatic athletes [13,30].

## 6. T-Wave Inversion in Pediatric Athletes

### 6.1. Definition and Clinical Relevance

TWI observed in pediatric athletes may reflect distinct physiological or pathological mechanisms, including age-related repolarization patterns, training-related electrical remodeling, and early manifestations of cardiomyopathy.

The presence of TWI represents for sports cardiology specialists one of the most challenging ECG findings in young athletes. TWI is defined as a negative T-wave ≥ 1 mm in depth in ≥2 contiguous leads, excluding leads III, aVR, and V1, according to current international ECG interpretation criteria for athletes, and should prompt further evaluation depending on distribution and associated features [13].

In pediatric populations, anterior TWI confined to leads V1–V3 in individuals under 16 years of age may reflect a physiological juvenile pattern related to incomplete right ventricular repolarization maturation [9]. However, lateral or inferolateral TWI is consistently considered abnormal and associated with increased likelihood of structural cardiomyopathy [15].

In addition to anterior and lateral patterns, isolated inferior T-wave inversion represents a relatively common finding in athletes and is generally considered to have a more benign clinical significance compared to other T-wave inversion distributions. When present in isolation and in the absence of symptoms, family history, or structural abnormalities, inferior T-wave inversion is less strongly associated with underlying cardiomyopathy [34].

However, as with other repolarization abnormalities, clinical context remains essential, and further evaluation may be warranted in the presence of additional risk markers.

### 6.2. Age- and Ethnicity-Related Patterns

Anterior TWI may persist during early adolescence and regress with maturation [9,35]. Persistence beyond mid-adolescence (≥16 years of age), extension beyond lead V2, or association with additional ECG abnormalities increases suspicion for underlying pathology. Notably, discrepancies between earlier Task Force Criteria and more recent international athlete-specific recommendations create a diagnostic gray zone in mid-adolescence (approximately 14–16 years of age). In this subgroup, anterior T-wave inversion extending beyond lead V2 may represent either a delayed physiological maturation pattern or the earliest manifestation of arrhythmogenic cardiomyopathy.

Therefore, ECG interpretation in this age range should not be considered in isolation. A comprehensive evaluation integrating clinical history, family history, and multimodality imaging is recommended. In selected cases, particularly when T-wave inversion extends beyond V2 or is associated with additional borderline findings, a cautious approach with periodic reassessment including echocardiography, exercise stress testing and 24 h Holter monitoring should be adopted to detect potential phenotypic evolution over time.

Ethnicity further modulates repolarization patterns. In Black athletes, anterior TWI preceded by convex ST-segment elevation and J-point elevation is often a benign physiological variant when isolated [8,14]. Recognition of this pattern is essential to reduce false-positive diagnoses and unnecessary restriction from competitive sport [8].

### 6.3. Longitudinal Evidence

Although the present review focuses on pediatric athletes, some landmark studies investigating T-wave inversion include broader cohorts of young individuals extending into early adulthood. These studies are discussed because they provide important insights into the clinical significance and outcomes of anterior T-wave inversion. Long-term follow-up studies in adolescent athletes suggest that isolated anterior TWI in V2–V3 is rarely associated with progression to structural heart disease when baseline imaging is normal [36,37,38]. Similarly, pediatric screening cohorts demonstrate that most cases of isolated anterior TWI with normal cardiac evaluation are not associated with a diagnosis of cardiomyopathy during follow-up [32].

However, lateral or inferolateral TWI remains strongly associated with cardiomyopathic substrates. Advanced imaging, particularly cardiac magnetic resonance (CMR), may identify subtle myocardial abnormalities in selected athletes with apparently isolated ECG findings [39]. Recent studies addressing T-wave inversion patterns in pediatric and adolescent athletes are summarized in Table 2.

### 6.4. Clinical Implications

Evaluation of TWI should include detailed clinical history, family history of cardiomyopathy or sudden death, transthoracic echocardiography, and when indicated, exercise testing and ambulatory ECG monitoring. The 2020 ESC Sports Cardiology Guidelines recommend comprehensive evaluation for lateral TWI and structured follow-up for selected anterior patterns in adolescents. Due to the potential for phenotypic evolution during adolescence, longitudinal reassessment remains essential to balance early disease detection with avoidance of unnecessary sports disqualification [16].

## 7. Differential Diagnosis and Red Flags

Ventricular repolarization abnormalities in pediatric athletes require careful differentiation between physiological adaptation to training and early manifestations of structural or electrical heart disease.

Hypertrophic cardiomyopathy (HCM) remains one of the leading causes of SCD in young athletes. Lateral TWI, pathological Q waves, and ST-segment depression increase suspicion for HCM and warrant comprehensive imaging assessment [40].

Arrhythmogenic cardiomyopathy (ACM) should be considered when anterior or inferolateral TWI persists beyond expected age limits (≥16 years of age) or when ventricular arrhythmias or a positive family history are present. Diagnosis should follow established Task Force Criteria integrating ECG, imaging, arrhythmic burden, and genetic findings [41].

Myocarditis may manifest as repolarization abnormalities, particularly when ECG modifications are associated with recent viral illness or elevated biomarkers (particularly cardiac troponin). In this context, CMR can play a central role in detecting myocardial inflammation and fibrosis [42].

Primary electrical diseases, including inherited channelopathies, may also manifest as repolarization abnormalities beyond isolated ERP or TWI patterns. QT interval abnormalities or polymorphic ventricular arrhythmias require targeted evaluation and specialist referral [43].

The ESC Guidelines emphasize that lateral TWI, complex ventricular arrhythmias, unexplained syncope, or abnormal imaging findings constitute red flags requiring advanced diagnostic work-up prior to sports participation [44]. Key electrocardiographic red flags suggesting underlying cardiac disease in pediatric athletes are summarized in Table 3.

## 8. Clinical Management and Longitudinal Follow-Up

Management of ventricular repolarization abnormalities in pediatric athletes should follow a structured and individualized approach aimed at distinguishing physiological adaptation from early disease while minimizing unnecessary restrictions, as summarized in Table 4.

**Figure 1:**
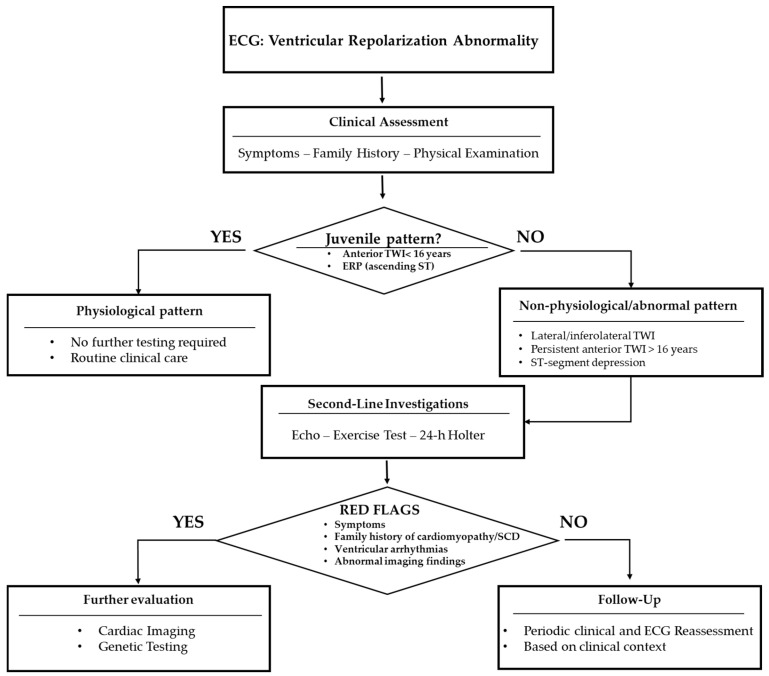
Proposed clinical algorithm for the evaluation of ventricular repolarization abnormalities in pediatric athletes. Abbreviations: ECG, electrocardiogram; ERP, early repolarization pattern; TWI, T-wave inversion; Echo, transthoracic echocardiography; 24 h Holter, 24 h ambulatory electrocardiographic monitoring.

Initial evaluation includes confirmation of ECG findings and adherence to current international standards for ECG interpretation [13]. A clinical algorithm for the evaluation of ventricular repolarization abnormalities in pediatric athletes proposed by the authors is illustrated in Figure 1. When abnormalities are atypical—such as persistent anterior TWI beyond adolescence (≥16 years of age) or lateral TWI—first-line investigations include transthoracic echocardiography, exercise testing, and ambulatory ECG monitoring. Current ventricular arrhythmia guidelines emphasize integrated risk stratification combining ECG morphology, arrhythmic burden, and imaging findings [44].

When suspicion persists, CMR provides incremental diagnostic value, particularly for detection of myocardial fibrosis or subtle cardiomyopathic phenotypes [45,46]. Tissue characterization with late gadolinium enhancement improves differentiation between physiological remodeling and pathological substrates.

Structured evaluation pathways for athletes with abnormal ECG findings support appropriate escalation of testing while avoiding unnecessary investigations [43]. Temporary restriction from competitive sport may be appropriate during ongoing evaluation in athletes with concerning features.

Although the present review focuses on pediatric athletes, similar electrocardiographic findings in adult athletes are generally managed according to established sports cardiology guidelines. In this population, evaluation of ventricular repolarization abnormalities similarly relies on integrated clinical assessment, ECG interpretation according to athlete-specific criteria, and stepwise use of imaging and functional testing to exclude cardiomyopathic substrates. Eligibility and return-to-play decisions are guided by current recommendations emphasizing individualized risk stratification and shared decision-making [15,47].

Due to the dynamic nature of cardiac maturation during adolescence, longitudinal follow-up with serial ECG and imaging reassessment remains central to safe management. This approach allows identification of phenotypic ECG evolution while supporting continued athletic participation when evaluation remains reassuring.

## 9. Limitations

This review has several limitations that should be acknowledged. First, it represents a narrative synthesis rather than a systematic review or meta-analysis. Although the literature search was structured and focused on clinically relevant studies, such selection bias cannot be entirely excluded.

Moreover, data specifically addressing ventricular repolarization abnormalities in strictly pediatric cohorts remain limited compared to adult athletic populations. Many available studies include mixed adolescent and young adult cohorts, which may limit direct extrapolation to younger age groups.

Then, longitudinal follow-up data in pediatric athletes with isolated early repolarization or anterior T-wave inversion remain relatively poor. While available evidence suggests a largely benign clinical course in the absence of additional risk markers, long-term prospective data extending into adulthood are still needed.

Finally, heterogeneity in study design, ECG definitions, and imaging protocols across published cohorts may limit direct comparison between studies.

Despite these limitations, the integration of current ECG interpretation standards, imaging advances, and emerging longitudinal data provides a practical framework for clinical decision-making in young athletes.

## 10. Conclusions

Ventricular repolarization abnormalities are common findings in pediatric athletes and most often reflect physiological adaptation to training combined with age-dependent cardiovascular maturation. Early repolarization pattern and anterior T-wave inversion frequently represent benign variants when interpreted according to current athlete-specific criteria [13] and within the appropriate clinical context.

However, lateral or inferolateral T-wave inversion, atypical ST-segment morphology, associated clinical symptoms, positive family history for cardiomyopathy or sudden cardiac death, and abnormal imaging findings should raise suspicion for underlying structural or electrical disease and need prompt comprehensive evaluation.

A structured, risk-based approach integrating ECG morphology, demographic context, imaging findings, and longitudinal follow-up is essential in this population. This strategy allows clinicians to identify cardiomyopathy at an early stage while limiting unnecessary diagnostic testing and preventing inappropriate sports restrictions.

Future research should focus on long-term prospective pediatric cohorts to better define the natural history and prognostic significance of ventricular repolarization abnormalities during the transition from adolescence to adulthood. Improved phenotypic characterization and integration of imaging and genetic data may further refine risk stratification in young athletes.

## Figures and Tables

**Table 1:** Characteristics of studies on early repolarization pattern (ERP) in pediatric athletes included in the review.

Study	Population	(N°)	ERP Definition	Key Findings	Follow-Up/Outcomes
Halasz et al. [30]	Pediatric athletes aged 7–16 y.o. undergoing pre-participation screening	886	J-point elevation ≥ 0.1 mV in ≥2 contiguous inferior and/or lateral leads	ERP prevalence ~13%; predominance of inferolateral distribution and notching morphology; dynamic phenotype over time	Median follow-up ~4 years; no major cardiovascular or arrhythmic events
Vecchiato et al. [32]	Adolescent competitive athletes aged 10–18 y.o.	600	J-point elevation ≥ 0.1 mV in inferior and/or lateral leads	ERP prevalence ~27%; sex-related differences in ST morphology (ascending more common in males)	No significant arrhythmias during maximal exercise testing
Çetin et al. [33]	Male teenage competitive athletes (10–18 years)	159	Standard ERP criteria (J-point elevation ≥ 0.1 mV)	Association between ERP and physiological left ventricular remodeling; no evidence of structural pathology	Cross-sectional study; no adverse clinical correlations reported

**Table 2:** Characteristics of studies on T-wave inversion (TWI) in pediatric athletes included in the review. Abbreviations: CMR: cardiac magnetic resonance.

Study	Population	ECG Pattern	Key Findings	Clinical Recommendations
Papadakis et al. [9]	Young athletes (14–18 years)	Anterior TWI (V1–V3)	Juvenile pattern may represent physiological variant in younger athletes	Interpret in context of age and pubertal status
Calore et al. [10]	Athletes with anterior TWI (predominantly Caucasian cohort)	Anterior TWI with J-point/ST elevation	Ethnicity-related physiological variant when isolated	Avoid unnecessary restriction when no additional abnormalities are present
Orchard et al. [37]	Elite adolescent athletes (14–18 years)	Isolated anterior TWI (V2–V3)	No progression to structural disease over ~6 years follow-up	Support conservative follow-up strategy
D’Ascenzi et al. [38]	Athletes with T-wave inversion undergoing CMR	Lateral/inferolateral TWI	Higher association with structural cardiomyopathy; CMR adds diagnostic value	Advanced imaging recommended when lateral TWI present
Malhotra et al. [36]	Young individuals (16–35 years), including athletes	Anterior TWI (mostly V1–V2)	Majority of cases confined to V1–V2 and not associated with cardiomyopathy	Anterior TWI limited to V1–V2 may represent a physiological variant in young individuals

**Table 3:** ECG red flags suggesting underlying cardiac disease in pediatric athletes. Abbreviations: ECG: electrocardiogram; Echo: transthoracic echocardiography; CMR: cardiac magnetic resonance; Holter: ambulatory electrocardiographic monitoring.

ECG Feature	Clinical Concern	Suggested Action
Lateral or inferolateral T-wave inversion	Suspected structural heart disease	Second-line evaluation (Echo, exercise test, Holter) → Consider CMR if abnormalities persist or suspicion remains
ST-segment depression	Suspected structural heart disease	Second-line evaluation (Echo, exercise test, Holter) → Consider CMR (for structural/functional assessment) or coronary imaging (for coronary anatomy) if abnormalities persist or suspicion remains
Pathological Q waves	Suspected structural heart disease	Echo → Consider CMR for further structural characterization if abnormalities are detected or suspicion persists
QT prolongation/shortening	Suspected channelopathy	Specialist referral ± genetic testing
Complex ventricular arrhythmias	Suspected cardiomyopathy	Second-line evaluation (Echo, exercise test, Holter) → Consider CMR if abnormalities persist or suspicion remains

**Table 4:** Structured clinical approach to ventricular repolarization abnormalities. Abbreviations: ECG, electrocardiogram; ERP, early repolarization pattern; TWI, T-wave inversion; Echo, transthoracic echocardiography; Holter, ambulatory electrocardiographic monitoring.

ECG Pattern	Initial Work-Up	Follow-Up Strategy
Typical ERP (ascending ST)	Clinical evaluation	No further testing required if isolated and in the absence of symptoms or family history, or other abnormal findings; routine clinical care
Anterior TWI < 16 years	Clinical evaluation	No further testing required if isolated and in the absence of symptoms or family history, or other abnormal findings; routine clinical care
Persistent anterior TWI ≥ 16 years	Echo + exercise test + Holter	Periodic follow-up with repeat evaluation; consider further imaging if abnormalities persist
Lateral/inferolateral TWI	Echo + exercise test + Holter	Strong consideration for CMR; additional testing (including genetic evaluation) based on findings

## Data Availability

No new data were created or analyzed in this study. Data sharing is not applicable to this article.

## Abbreviations

The following abbreviations are used in this manuscript:

SCD	Sudden cardiac death
ECG	Electrocardiogram
VRAs	Ventricular repolarization abnormalities
ERP	Early repolarization pattern
TWI	T-wave inversion
CMR	Cardiac magnetic resonance
